# Legal Notes for Institutional Workers

**Published:** 1912-03-23

**Authors:** 


### LEGAL NOTES FOR INSTITUTIONAL WORKERS.

**(The Editor will be pleased to answer Legal queries in this column.)**

### Hospital Management and the Law of Defamation.

A slander action which was recently tried in the King's Bench Division is not without its lesson to the governors of an institution in their relation to the staff. The general manager of an insurance company brought an action for damages against one of his directors on the ground that, at a directors' meeting, the defendant accused him of "telling a deliberate falsehood" and "throwing dust in the eyes of the directors." The jury found that the words were untrue, but that they were spoken by the defendant without malice and in the honest discharge of his duties as a director. He therefore had judgment with costs. The position of a hospital governor or committee man may be likened to that of a company director. Just as a director has to look after the interests of the shareholders the responsible heads of an institution are the servants of the subscribers. They have also to consider the welfare of the patients; and it would be a serious matter if every piece of adverse criticism could be made the subject of an action for slander. A former judge of the King's Bench Division was fully warranted when he said: "The doctrine of privilege has been made part and parcel of our law for the common convenience of society."

### Testimonials to Character.

A case which has been reported in the newspapers draws attention to a matter which the employer who is asked to give a character should bear in mind. A man whose name was given as a reference stated that an assistant who had been in his employment for three years was honest and a good worker. On this recommendation the assistant obtained a new situation. He turned out to be dishonest. His new employer brought an action for damages against the "reference." It was apparently proved at the hearing that when he gave the character the defendant knew that another employer had alleged dishonesty against the man, but did not reveal this fact. He had to pay £21 damages. A case of this kind emphasises the necessity for absolute candour when giving a character.

The giving and "taking up" of references are things of daily occurrence at every large hospital. When there is nothing but good to be said of a nurse or a servant, it is a pleasure to write a character. It is otherwise when the conduct of the party seeking a note of commendation has been unsatisfactory.

### Testimonials and the Law.

Omitting the method unsuccessfully adopted in the above case, there are two ways of escape. A character may be refused or may be made colourless; but to give a character which is wholly colourless is almost as prejudicial to the employee as refusal to give any character at all. Moreover it is doubtful whether anyone can ever be justified in refusing to state all he knows about persons who are seeking employment at a hospital. Having regard to these considerations, it is important to notice that the law affords considerable protection to him who is asked to give a character. No action for libel or slander can be founded on a character, provided the person who gives it is not actuated by malice. According to Dr. Odgers in his *Law of Libel and Slander*: "A duty is cast upon the former master to state fully and honestly all that he knows either for or against the servant, and any communication made in the performance of this duty is clearly privileged for the sake of the common convenience of society, even though it should turn out that the former master was mistaken in some of his statements. But if the master, knowing that the servant deserves a good character, yet, having some grudge against him, or from some other malicious motive, deliberately states what he knows to be false and gives his late servant a bad character, then such a communication is not a performance of the duty and therefore is not privileged. There is, in fact, in such a case, evidence of malice which takes the case out of privilege." It is well to bear in mind that a letter written by an employer to retract a favourable character given previously will also be privileged.

